# Supragingival Plaque Microbiome Ecology and Functional Potential in the Context of Health and Disease

**DOI:** 10.1128/mBio.01631-18

**Published:** 2018-11-27

**Authors:** Josh L. Espinoza, Derek M. Harkins, Manolito Torralba, Andres Gomez, Sarah K. Highlander, Marcus B. Jones, Pamela Leong, Richard Saffery, Michelle Bockmann, Claire Kuelbs, Jason M. Inman, Toby Hughes, Jeffrey M. Craig, Karen E. Nelson, Chris L. Dupont

**Affiliations:** a Department of Microbial and Environmental Genomics, J. Craig Venter Institute, La Jolla, California, USA; b Departments of Human Biology and Genomic Medicine, J. Craig Venter Institute, Rockville, Maryland, USA; c Departments of Human Biology and Genomic Medicine, J. Craig Venter Institute, La Jolla, California, USA; d Human Longevity Institute, La Jolla, California, USA; e Murdoch Children’s Research Institute and Department of Pediatrics, University of Melbourne, Royal Children’s Hospital, Parkville, VIC, Australia; f School of Dentistry, The University of Adelaide, Adelaide, SA, Australia; g Centre for Molecular and Medical Research, School of Medicine, Deakin University, Geelong, VIC, Australia; VA Palo Alto Health Care System

**Keywords:** *Streptococcus*, metabolism, metagenomics, microbial ecology, oral microbiology

## Abstract

To address the question of how microbial diversity and function in the oral cavities of children relates to caries diagnosis, we surveyed the supragingival plaque biofilm microbiome in 44 juvenile twin pairs. Using shotgun sequencing, we constructed a genome encyclopedia describing the core supragingival plaque microbiome. Caries phenotypes contained statistically significant enrichments in specific genome abundances and distinct community composition profiles, including strain-level changes. Metabolic pathways that are statistically associated with caries include several sugar-associated phosphotransferase systems, antimicrobial resistance, and metal transport. Numerous closely related previously uncharacterized microbes had substantial variation in central metabolism, including the loss of biosynthetic pathways resulting in auxotrophy, changing the ecological role. We also describe the first complete *Gracilibacteria* genomes from the human microbiome. Caries is a microbial community metabolic disorder that cannot be described by a single etiology, and our results provide the information needed for next-generation diagnostic tools and therapeutics for caries.

## Abstract (precis)

Oral health has substantial economic importance, with over $100 billion spent on dental care in the United States annually. The microbiome plays a critical role in oral health, yet remains poorly classified. To address the question of how microbial diversity and function in the oral cavities of children relate to caries diagnosis, we surveyed the supragingival plaque biofilm microbiome in 44 juvenile twin pairs. Using shotgun sequencing, we constructed a genome encyclopedia describing the core supragingival plaque microbiome. This unveiled several new previously uncharacterized but ubiquitous microbial lineages in the oral microbiome. Caries is a microbial community metabolic disorder that cannot be described by a single etiology, and our results provide the information needed for next-generation diagnostic tools and therapeutics for caries.

## INTRODUCTION

The oral microbiome is a critical component of human health. There are an estimated 2.4 billion cases of untreated tooth decay worldwide, making the study of carious lesions, and their associated microbiota, a topic of utmost importance from the interest of public health (1). Oral disease poses a considerable socioeconomic burden within the United States; the national dental care expenditure exceeded $113 billion in 2014 (2). Despite the widespread impact of oral diseases, much of the oral microbiome is poorly characterized; of approximately 700 species identified (3), 30% have yet to be cultivated (4). On average, less than 60% of oral metagenomic reads can be mapped to reference databases at species-level specificity (5). The characterization of functional potential plasticity, (intra/inter)organismal interactions, and responses to environmental stimuli in the oral microbiomes are essential in interpreting the complex narratives that orchestrate host phenotypes.

Oral microbiomes are important not only in the immediate environment of the oral cavity, but also systemically as well. For instance, although dental caries, the most common chronic disease in children (6), is of a multifactorial nature, it usually occurs when sugar is metabolized by acidogenic plaque-associated bacteria in biofilms, resulting in increased acidity and dental demineralization, a condition exacerbated by frequent sugar intake (7). Similarly, in periodontitis, a bacterial community biofilm (plaque) elicits local and system inflammatory responses in the host, leading to the destruction of periodontal tissue, pocket formation, and tooth loss (8, 9). Likewise, viruses and fungi in oral tissues can trigger gingival lesions associated with herpes and candidiasis (10) in addition to cancerous tissue in oral cancer (11). The connections between oral microbes and health extend beyond the oral cavity as cardiometabolic, respiratory, and immunological disorders and some gastrointestinal cancers are thought to have associations with oral microbes (12–15). For example, the enterosalivary nitrate cycle originates from dissimilatory nitrite reduction in the oral cavity but influences nitric oxide production throughout the body (16). Consequently, unraveling the forces that shape the oral microbiome is crucial for the understanding of both oral and broader systemic health.

Streptococcus mutans has been the focal point of research involving cariogenesis and the associated dogma since the species discovery in 1924 (17). However, prior to the characterization of S. mutans, dental caries etiology was believed to be community driven rather than the product of a single organism (18). There is no question that S. mutans can be a driving factor in cariogenesis; S. mutans colonized onto sterilized teeth form caries (17). However, the source of the precept transpired from an era inherently subject to culturing bias. Mounting evidence exists supporting the claim that S. mutans is not the sole pathogenic agent responsible for carious lesions (19–25) with characterization of oral biofilms by metabolic activity and taxonomic composition rather than only listing dominant species (26). A recent large-scale 16S rRNA gene survey of juvenile twins by our team found that while there are heritable microbes in supragingival plaque, the taxa associated with the presence of caries are environmentally acquired and seem to displace the heritable taxa with age (27). Another surprising facet of this recent study was the lack of a statistically robust association between the relative abundance of S. mutans and the presence of carious lesions.

The nature of the methods used in that recent 16S rRNA amplicon study addressed questions related to community composition but did provide the means to examine links between microbiome phylogenetic diversity, functional potential, and host phenotype. Here, we characterize the microbiome in supragingival biofilm swabs of monozygotic (MZ) and dizygotic (DZ) twin pairs, including children both with and without dental caries, using shotgun metagenomic sequencing, coassembly, and binning techniques.

## RESULTS

### Data overview.

The supragingival microbiomes of 44 twin pairs were sampled via tooth swab, with the twin pairs being chosen based on 16S rRNA data (see “Study design” in Materials and Methods). Of the 88 samples, 50 (56.8%) were positive for caries. Shotgun sequencing of whole-community DNA was conducted to produce an average of 3 million fragments of non-human DNA per sample. Human reads, accounting for ∼20% of the total, were removed, and all data were subjected to metagenomics coassembly using metaSPAdes and subsequent quality control with QUAST (28). The contigs were annotated for phylogenetic and functional content and then binned by *k*-mer utilization and across sample representation into genome bins. Thirty-four of these passed quality control and were graduated to metagenome assembled genomes (MAGs) (Table 1; see Table S5 in the supplemental material; see also Fig. 5). MAGs are sets of assembled contigs determined to likely originate from the same organism based on basic nucleotide usage (e.g., *k*-mer) or coverage across different sample sets; there are established methods for determining if these are pure (29) or complete (30). These are likely to not capture genomic “island regions” that are specific to individual samples but can be considered a consensus core genome for a specific species (31). Contigs annotated as *Streptococcus* interfered with the *k*-mer binning but were also easily identified; therefore, we removed these contigs to facilitate the binning of other genomes. The *Streptococcus* population was examined using the pangenomic analysis package MIDAS (5). Reads from each subject were mapped back to the genome bins to create an assembly-linked matrix of relative abundance, phylogenetically characterized genome origin, genome bin functional contents, variance component estimation, and the phenotypic state of the host (caries or not).

**TABLE 1:** Microbial community description^*a*^

Identifier	Taxonomy				CheckM		Variance component estimation			Genome		
	Family	Genus	Species	Group	Completeness	Contamination	A	C	E	Size (nt)	GC content	No. of ORFs
bin_1	*Actinomycetaceae*	*Actinomyces*	*Actinomyces* sp. oral taxon 178	I.A	74.29	4.44	0.72288	0	0.27712	1,799,133	0.715138347	1,877
bin_2	*Actinomycetaceae*	*Actinomyces*	*Actinomyces* sp. oral taxon 848	I.B	78.45	0	0.52721	0.28455	0.18824	1,541,794	0.69676818	1,710
bin_3	*Prevotellaceae*	*Alloprevotella*	Alloprevotella rava	II.A	89.42	5.33	0.63739	0	0.36261	2,086,006	0.549467739	2,818
bin_4	*Prevotellaceae*	*Alloprevotella*	Alloprevotella rava	II.B	89.51	44.75	0.22208	0.31498	0.46293	3,203,447	0.473549274	4,755
bin_5	Unclassified *Bacteroidetes*	Unclassified *Bacteroidetes*	*Bacteroidetes* oral taxon 274		96.55	40.66	0.64422	0	0.35578	2,486,193	0.434083649	3,992
bin_6	*Campylobacteraceae*	*Campylobacter*	Campylobacter concisus		49.92	0	0.21324	0.26944	0.51732	1,200,135	0.374629521	2,002
bin_7	*Campylobacteraceae*	*Campylobacter*	Campylobacter gracilis		85.89	55.88	0.68857	0	0.31143	3,163,899	0.460077898	4,920
bin_8	Unclassified *Saccharibacteria*	“*Candidatus* Saccharimonas”	“*Candidatus* Saccharimonas aalborgensis”	III.A	86.21	159.28	0.6728	0	0.3272	2,811,149	0.46770591	4,728
bin_9	Unclassified *Saccharibacteria*	“*Candidatus* Saccharimonas”	“*Candidatus* Saccharimonas aalborgensis”	III.B	74.84	9.95	0.55187	0.14287	0.30526	596,323	0.353912225	1,065
bin_10	Unclassified *Saccharibacteria*	“*Candidatus* Saccharimonas”	“*Candidatus* Saccharimonas aalborgensis”	III.C	65.91	14.11	0.7714	0	0.2286	1,065,796	0.522207995	1,829
bin_11	*Flavobacteriaceae*	*Capnocytophaga*	Capnocytophaga gingivalis		85.34	207.14	0.43743	0.06552	0.49705	7,550,959	0.413623753	11,576
bin_12	*Cardiobacteriaceae*	*Cardiobacterium*	Cardiobacterium hominis		73.46	49.06	0.17702	0	0.82298	3,863,328	0.596540599	5,759
bin_13	*Lachnospiraceae*	*Catonella*	Catonella morbi		72.41	28.29	0.2301	0.44126	0.32865	2,490,472	0.444225432	4,105
bin_14	*Corynebacteriaceae*	*Corynebacterium*	Corynebacterium matruchotii		89.34	3.45	0.34153	0.28628	0.37219	2,342,592	0.577838138	3,442
bin_15	Unclassified *Gracilibacteria*	Unclassified *Gracilibacteria*	*Gracilibacteria* bacterium JGI 0000069-K10	IV.A	79.31	56.66	0	0.20796	0.79204	1,663,606	0.370415832	1,658
bin_16	Unclassified *Gracilibacteria*	Unclassified *Gracilibacteria*	*Gracilibacteria* bacterium JGI 0000069-K10	IV.B	93.1	88.17	0	0.17557	0.82443	1,991,479	0.249330272	2,026
bin_17	*Carnobacteriaceae*	*Granulicatella*	Granulicatella elegans		86.21	45.96	0.37126	0	0.62874	2,163,485	0.347674701	3,562
bin_18	*Neisseriaceae*	*Kingella*	Kingella denitrificans		79.95	3.45	0.12324	0.19897	0.6778	1,689,409	0.56719184	2,733
bin_19	*Neisseriaceae*	*Kingella*	Kingella oralis		66.22	5.17	0.1973	0.02227	0.78043	1,716,807	0.54631534	2,995
bin_20	*Lachnospiraceae*	*Lachnoanaerobaculum*	Lachnoanaerobaculum saburreum		81.9	0	0	0.00123	0.99877	1,629,684	0.401290066	2,758
bin_21	*Lachnospiraceae*	Unclassified *Lachnospiraceae*	*Lachnospiraceae* bacterium oral taxon 500		95.69	243.39	0.69892	0	0.30108	10,502,342	0.35810365	16,794
bin_22	*Burkholderiaceae*	*Lautropia*	Lautropia mirabilis		93.97	22.57	0.48924	0	0.51076	4,240,867	0.658203853	5,584
bin_23	*Leptotrichiaceae*	*Leptotrichia*	Leptotrichia buccalis		68.13	1.72	0.60264	0	0.39736	1,129,771	0.290444701	1,818
bin_24	*Moraxellaceae*	*Moraxella*	Moraxella catarrhalis		100	16.09	0.66539	0	0.33461	2,551,229	0.381399318	4,353
bin_25	*Neisseriaceae*	*Neisseria*	Neisseria oralis		84.5	176.68	0.6901	0	0.3099	7,906,650	0.506099296	13,143
bin_26	*Porphyromonadaceae*	*Porphyromonas*	Porphyromonas gingivalis		79.31	0	0.4859	0	0.5141	1,549,578	0.427495099	2,319
bin_27	*Prevotellaceae*	*Prevotella*	Prevotella oulorum		85.11	1.03	0.48539	0	0.51461	2,201,265	0.481471336	3,248
bin_28	*Prevotellaceae*	*Prevotella*	Prevotella pallens		69.83	25.24	0.58443	0	0.41557	2,593,194	0.394058832	4,155
bin_29	*Prevotellaceae*	*Prevotella*	*Prevotella* sp. oral taxon 472	V.A	69.51	20.69	0.5945	0	0.4055	4,133,493	0.470396587	6,459
bin_30	*Prevotellaceae*	*Prevotella*	*Prevotella* sp. oral taxon 472	V.B	69.69	1.72	0.70867	0	0.29133	2,023,841	0.430838687	3,203
bin_31	*Flavobacteriaceae*	*Riemerella*	Riemerella anatipestifer		79	12.07	0.60062	0	0.39938	1,754,497	0.378369983	2,916
bin_32	*Streptococcaceae*	*Streptococcus*	*Streptococcus* pangenome		99.22	288.36	0.09391	0.59629	0.3098	12,438,952	0.382878879	21,444
bin_33	*Tannerellaceae*	*Tannerella*	Tannerella forsythia		80.09	1.72	0.53162	0	0.46838	2,246,045	0.587740228	2,959
bin_34	*Veillonellaceae*	*Veillonella*	*Veillonella* sp. oral taxon 780		91.07	91.08	0.1678	0.06556	0.76664	3,219,265	0.387835111	5,471

a Recovered draft genomes with taxonomy, identifier mapping, CheckM statistics, variance component estimation (i.e., ACE model), and genome statistics.

### Core supragingival microbiome community, phenotype-specific taxonomic enrichment, and cooccurrence.

**FIG 1:**
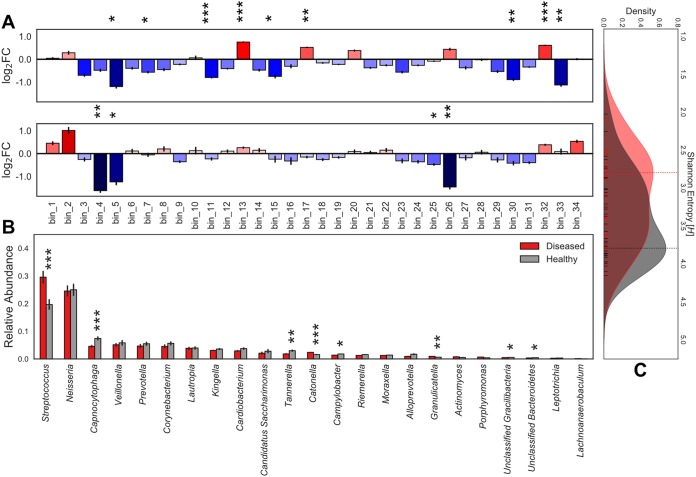
Core supragingival microbiome composition in the context of health and disease. Microbial community abundance profiles and enrichment in phenotype-specific cohorts. Statistical significance (*P* < 0.001, ***; *P* < 0.01, **; and *P* < 0.05, *). Error bars represent SEM. (A) Mean of pairwise log_2_ fold changes between phenotype subsets. (Top) Caries-positive versus caries-negative individuals with red indicating enrichment of taxa in the caries-positive cohort. (Bottom) Subjects with caries that has progressed to the dentin layer versus enamel-only caries with blue denoting taxa enriched in the enamel. (B) Relative abundance of TPM values for each genera of *de novo* assembly grouped by caries-positive and caries-negative cohorts. *Actinomyces* = 0.36, *Alloprevotella* = 0.0515, *Campylobacter* = 0.0124, “*Candidatus* Saccharimonas” = 0.107, *Capnocytophaga* = 0.000875, *Cardiobacterium* = 0.0621, *Catonella* = 0.000264, *Corynebacterium* = 0.0552, *Granulicatella* = 0.00255, *Kingella* = 0.252, *Lachnoanaerobaculum* = 0.0791, *Lautropia* = 0.279, *Leptotrichia* = 0.0829, *Moraxella* = 0.215, *Neisseria* = 0.455, *Porphyromonas* = 0.136, *Prevotella* = 0.12, *Riemerella* = 0.0621, *Streptococcus* = 0.000901, *Tannerella* = 0.0061, unclassified *Bacteroidetes* = 0.0111, unclassified *Gracilibacteria* = 0.0316, *Veillonella* = 0.471. (C) Kernel density estimation of Shannon entropy alpha diversity distributions for caries-positive (red) and caries-negative (gray) subjects calculated from normalized core supragingival community composition. Vertical lines indicate the mode of the kernel density estimate distributions for each cohort. Statistical significance (*P* = 0.0109).

We observed a genome-resolved microbiome dominated by *Streptococcus* and *Neisseria* across the cohort with relatively little variability (Fig. 1). Many of the taxa previously associated with supragingival biofilms were found (4), but we report the first binned genomes for *Gracilibacteria,* which have only been previously observed in the oral cavity using 16S rRNA screens. Three genomes for the TM7 lineage (32–34) were also recovered with substantial differences in genome architecture. Each of the 88 subjects contains the same genomes; these genomes comprise a core supragingival plaque microbiome that has relatively minor but statistically significant changes in proportional abundance. Sequences and genome bins for Archaea, Eukaryota (other than the host), or viruses were not observed. The core supragingival plaque microbiome defined here certainly includes far more than the 34 genome bins and 119 *Streptococcus* strains recovered here, though they are by nature numerically rare members of the community. These rare organisms could be later captured using deeper sequencing and a combination of reference-based and assembly-centric binning in an iterative manner.

**FIG 2:**
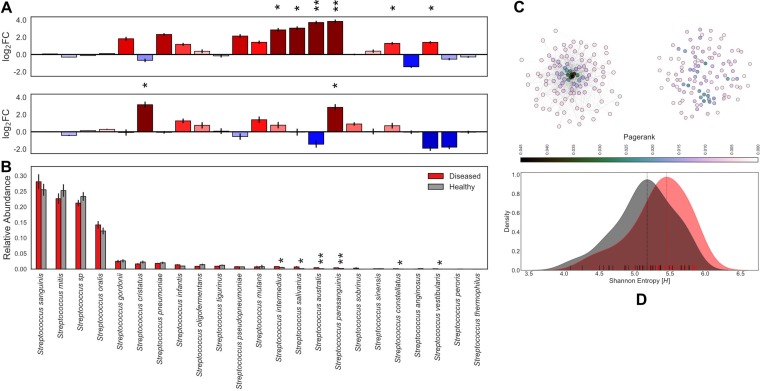
*Streptococcus* community composition in the context of health and disease. *Streptococcus* community abundance analysis and enrichment in phenotype-specific cohorts. Statistical significance (*P* < 0.001, ***; *P* < 0.01, **; and *P* < 0.05, *). Error bars represent SEM. (A) Mean of pairwise log_2_ fold changes between phenotype subsets. (Top) Caries-positive versus caries-negative individuals with red indicating enrichment of taxa in the caries-positive cohort. (Bottom) Subjects with caries that has progressed to the dentin layer versus enamel-only caries with blue denoting taxa enriched in the enamel. Pseudocount of 1e−4 applied to entire-count matrix for log transformation. (B) Relative abundance of MIDAS counts for each *Streptococcus* species grouped by caries-positive (red) and caries-negative (gray) subjects. (C) Fully connected undirected networks for diseased (right) and healthy (left) groups separately. Edge weights represent topological overlap measures, nodes colored by PageRank centrality, and the Fruchterman-Reingold force-directed algorithm for the network layout. (D) Kernel density estimation of Shannon entropy alpha diversity distributions for caries-positive (red) and caries-negative (gray) subjects calculated from normalized Streptococcus community composition. Vertical lines indicate the mode of the kernel density estimate distributions for each cohort. Statistical significance (*P* = 0.008).

The presence of caries is associated with statistically significant enrichments in the relative abundance of specific taxa when comparing the caries-negative and caries-positive cohorts (Fig. 1B). Namely, the abundance profiles of *Streptococcus*, Catonella morbi, and Granulicatella elegans were enriched in caries-positive subjects, while Tannerella forsythia, *Gracilibacteria*, Capnocytophaga gingivalis, *Bacteroides* sp. oral taxon 274, and Campylobacter gracilis were more abundant in the healthy cohort (Fig. 1A; Table S2). Community composition profiles were different according to caries progression; Alloprevotella rava, Porphyromonas gingivalis, Neisseria oralis, and *Bacteroides* oral taxon 274 were significantly enriched in subjects with carious enamel only (Fig. 1A). The *Streptococcus* community across all microbiomes contained 119 strain genomes detected at high resolution (Fig. 2). Within this mixed *Streptococcus* community, 86% is composed of S. sanguinis, S. mitis, S. oralis, and an unnamed *Streptococcus* sp. As with the bulk community, we found statistically significant strain abundance variations associated with the presence of caries in S. parasanguinis, S. australis, S. salivarius, S. intermedius, S. constellatus, and S. vestibularis; this enrichment was specific to rare strains that make up less than 5% of the *Streptococcus* (Fig. 2A and B; Table S2). During the global progression of carious lesions from enamel to dentin, we observed a modest statistical enrichment (*P < *0.05) in S. parasanguinis and S. cristatus (Table S2). However, these are general trends considering phenotype subsets for a single time point per individual; this data set does not support a longitudinal analysis, which will be the topic of future studies.

10.1128/mBio.01631-18.4 TABLE S1 Human subject metadata. Metadata for human subject used in the analysis. Download Table S1, TXT file, 0.02 MB. Copyright © 2018 Espinoza et al. 2018 Espinoza et al. This content is distributed under the terms of the Creative Commons Attribution 4.0 International license.

10.1128/mBio.01631-18.5 TABLE S2 Statistical significance for enrichment. MAG and *Streptococcus* species-level enrichment comparing caries-negative versus caries-positive and enamel-positive versus dentin-positive. Download Table S2, TXT file, 0.01 MB. Copyright © 2018 Espinoza et al. 2018 Espinoza et al. This content is distributed under the terms of the Creative Commons Attribution 4.0 International license.

To investigate the role of community alpha diversity in caries, we compared Shannon entropy measures for the healthy and diseased cohorts separately in both the core supragingival and strain-level *Streptococcus* communities. At the bulk community scale, healthy individuals (mode = 3.793 *H*) had statistically greater taxonomic diversity than the diseased subset (mode = 2.796 *H*) as shown in Fig. 1C. Adversely, the *Streptococcus* strain-level analysis revealed a statistical enrichment of diversity within the diseased cohort (mode = 5.452 *H*) compared to the healthy subset (mode = 5.177 *H*) as shown in Fig. 2D.

**FIG 3:**
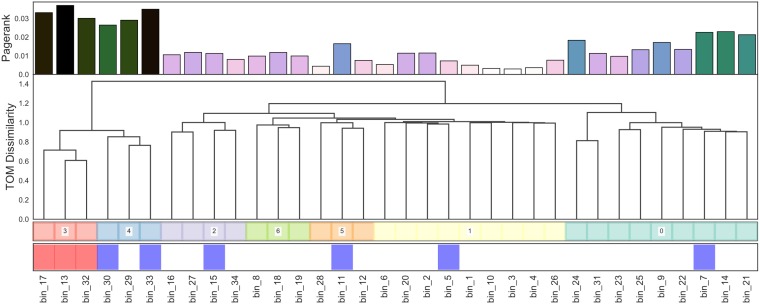
Core supragingival microbial cooccurrence network topology. Fully connected undirected cooccurrence network from normalized abundance profiles. (Dendrogram) Clustering of taxa using topological overlap dissimilarity and ward linkage with PageRank centrality (top row) and statistical enrichment in healthy or diseased cohorts. Statistical significance (*P* < 0.05; red, enriched in diseased; blue, enriched in healthy).

An examination of a genome cooccurrence network revealed a distinct topology illustrated in Fig. 3. For each genome, we also computed PageRank centrality, a variant of eigenvector centrality (35), which allows us to measure the influence of bacterial nodes within our cooccurrence networks. A select few MAGs form a cooccurrence cluster (Fig. 3; cluster 3, 4; *n* = 6) containing the 6 most highly connected taxa, 3 of which are statistically enriched in the diseased cohort while 2 are enriched in the healthy cohort. This microbial clique contains the highest-ranking PageRank centrality MAGs in the network and can be cleanly divided into a caries-affiliated subset and a subset enriched in bacteria associated with healthy phenotypes. The *Streptococcus* strain-level cooccurrence network topology varied greatly between caries-positive and -negative cohorts (Fig. 2C). Specifically, we observed that healthy subjects harbored a *Streptococcus* community influenced by a few key strains with high PageRank measures. In contrast to the healthy cohort, caries-affected individuals harbored a much more dispersed strain-level *Streptococcus* cooccurrence network.

### Phylogeny-linked functional profiles across caries phenotypes.

**FIG 4:**
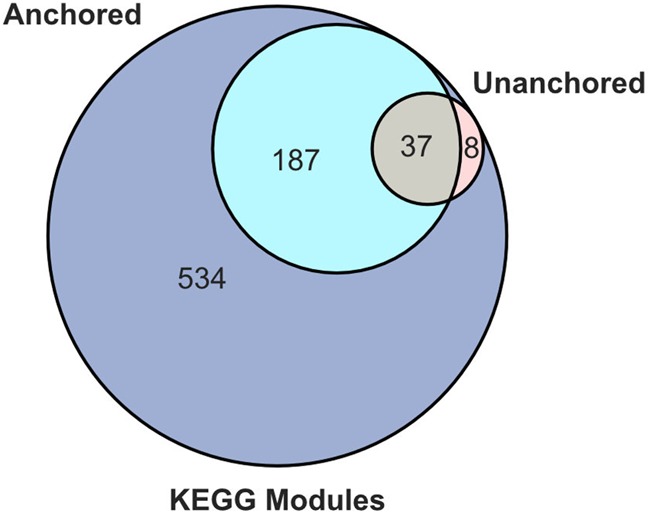
KEGG module significance to phenotype. Venn diagram of numbers of significant KEGG modules identified by unanchored and anchored approaches (*P* < 0.05).

To determine if caries presence is associated with trends in functional potential of the supragingival microbiome, we tested for functional enrichment in both a taxonomically anchored and unanchored fashion (Fig. 4). The “unanchored approach” simply tests whether KEGG modules are statistically enriched in the taxa statistically significant to caries status. The “anchored approach” combines the functional potential of a MAG, represented by a KEGG module completion ratio (MCR), with its normalized abundance values collapsing into a single phylogenomically binned functional potential (PBFP) profile. PBFP encodes information regarding genome size, community proportions, and functional potential for each sample which can be used in parallel with subject-specific metadata for machine learning and statistical methods. The overlap of these two methods identified 37 KEGG metabolic modules positively associated with caries-positive microbiomes (Fig. 4 and Table 2). Numerous phosphotransferase sugar uptake systems were more abundant in the caries-positive communities, including systems for the uptake of glucose (M00809, M00265, and M00266), galactitol (M00279), lactose (M00281), maltose (M00266), alpha glucoside (M00268), cellobiose (M00275), and *N*-acetylgalactosamine (M00277). Also positively associated with diseased host phenotypes was an increased abundance of numerous two-component histidine kinase-response regulator systems, including AgrC-AgrA (exoprotein synthesis) (M00495), BceS-BceR (bacitracin transport) (M00469), LiaS-LiaR (cell wall stress response) (M00481), and LytS-LytR (M00492) along with those putatively associated with virulence regulation such as SaeS-SaeR (staphylococcal virulence regulation) (M00468), Ihk-Irr (M00719), and ArlS-ArlR (M00716). Note that while these two-component systems have been characterized in specific lineages (e.g., Staphylococcus aureus), the KEGG modules detect their orthologs across diverse lineages. Modules associated with xenobiotic efflux pump transporters were enriched, including BceAB transporter (bacitracin resistance) (M00738), multidrug resistance EfrAB (M00706), multidrug resistance MdlAB/SmdAB (M00707), and tetracycline resistance TetAB(46) (M00635) along with antibiotic resistance, including cationic antimicrobial peptide (CAMP) resistance (M00725) and nisin resistance (M00754). Furthermore, numerous trace metal transport systems were enriched, including those for manganese/zinc (M00791, M00792), nickel (M00245, M00246), and cobalt (M00245).

**TABLE 2:** Metabolic modules significant to host phenotype^*a*^

KEGG module ID	Category	Type	Description	*P*, unanchored	*P*, anchored
M00495	Two-component regulatory system	FuncSet	AgrC-AgrA (exoprotein synthesis) two-component regulatory system	0.009495785	0.000617637
M00716	Two-component regulatory system	FuncSet	ArlS-ArlR (virulence regulation) two-component regulatory system	0.001647934	0.000458466
M00550	Cofactor and vitamin biosynthesis	Pathway	Ascorbate degradation, ascorbate→d-xylulose-5P	0.005276233	0.001411826
M00738	Drug efflux transporter/pump	FuncSet	Bacitracin resistance, BceAB transporter	0.011530602	0.000655047
M00469	Two-component regulatory system	FuncSet	BceS-BceR (bacitracin transport) two-component regulatory system	0.011530602	0.000655047
M00170	Carbon fixation	Pathway	C_4_-dicarboxylic acid cycle, phosphoenolpyruvate carboxykinase type	0.044692324	0.002118532
M00095	Terpenoid backbone biosynthesis	Pathway	C_5_ isoprenoid biosynthesis, mevalonate pathway	0.027543055	0.004677421
M00725	Drug resistance	Signature	Cationic antimicrobial peptide (CAMP) resistance, *dltABCD* operon	0.001647934	0.000458466
M00245	Metallic cation, iron siderophore, and vitamin B_12_ transport system	Complex	Cobalt/nickel transport system	0.038390231	0.001373416
M00045	Histidine metabolism	Pathway	Histidine degradation, histidine→*N*-formiminoglutamate→glutamate	0.047335834	0.014101382
M00375	Carbon fixation	Pathway	Hydroxypropionate-hydroxybutylate cycle	0.014395927	0.040233585
M00719	Two-component regulatory system	FuncSet	Ihk-Irr (virulence regulation) two-component regulatory system	0.009495785	0.000694538
M00481	Two-component regulatory system	FuncSet	LiaS-LiaR (cell wall stress response) two-component regulatory system	0.030844957	0.00106832
M00492	Two-component regulatory system	FuncSet	LytS-LytR two-component regulatory system	0.010611498	0.000927012
M00791	Metallic cation, iron siderophore, and vitamin B_12_ transport system	Complex	Manganese/zinc transport system	0.009523261	0.000715096
M00792	Metallic cation, iron siderophore, and vitamin B_12_ transport system	Complex	Manganese/zinc transport system	0.009523261	0.000826561
M00706	Drug efflux transporter/pump	Complex	Multidrug resistance, EfrAB transporter	0.009495785	0.000617637
M00707	Drug efflux transporter/pump	Complex	Multidrug resistance, MdlAB/SmdAB transporter	0.022443805	0.017060525
M00298	ABC-2-type and other transport systems	Complex	Multidrug/hemolysin transport system	0.047823524	0.000694538
M00205	Saccharide, polyol, and lipid transport system	Complex	*N*-Acetylglucosamine transport system	0.047823524	0.000850689
M00246	Metallic cation, iron siderophore, and vitamin B_12_ transport system	Complex	Nickel transport system	0.038390231	0.001373416
M00754	Drug resistance	FuncSet	Nisin resistance, phage shock protein homolog LiaH	0.030844957	0.00106832
M00277	Phosphotransferase system (PTS)	Complex	PTS system, *N*-acetylgalactosamine-specific II component	0.033036099	0.000875463
M00268	Phosphotransferase system (PTS)	Complex	PTS system, alpha-glucoside-specific II component	0.010611498	0.000472476
M00275	Phosphotransferase system (PTS)	Complex	PTS system, cellobiose-specific II component	0.037454277	0.000694538
M00279	Phosphotransferase system (PTS)	Complex	PTS system, galactitol-specific II component	0.00176648	0.000163547
M00287	Phosphotransferase system (PTS)	Complex	PTS system, galactosamine-specific II component	0.037454277	0.001619
M00809	Phosphotransferase system (PTS)	Complex	PTS system, glucose-specific II component	0.001647934	0.000458466
M00265	Phosphotransferase system (PTS)	Complex	PTS system, glucose-specific II component	0.010611498	0.000472476
M00281	Phosphotransferase system (PTS)	Complex	PTS system, lactose-specific II component	0.009523261	0.000900899
M00266	Phosphotransferase system (PTS)	Complex	PTS system, maltose/glucose-specific II component	0.008844291	0.000532576
M00603	Saccharide, polyol, and lipid transport system	Complex	Putative aldouronate transport system	0.011530602	0.000655047
M00589	Phosphate and amino acid transport system	Complex	Putative lysine transport system	0.011682199	0.000636088
M00468	Two-component regulatory system	FuncSet	SaeS-SaeR (staphylococcal virulence regulation) two-component regulatory system	0.001647934	0.000458466
M00633	Central carbohydrate metabolism	Pathway	Semi-phosphorylative Entner-Doudoroff pathway, gluconate/galactonate→glycerate-3P	0.011530602	0.005818074
M00635	Drug efflux transporter/pump	Complex	Tetracycline resistance, TetAB(46) transporter	0.009495785	0.000617637
M00159	ATP synthesis	Complex	V/A-type ATPase, prokaryotes	0.020814043	0.000736214

a KEGG modules significant in both anchored and unanchored approaches with hierarchical descriptions.

### Metabolic tradeoffs between closely related species.

**FIG 5:**
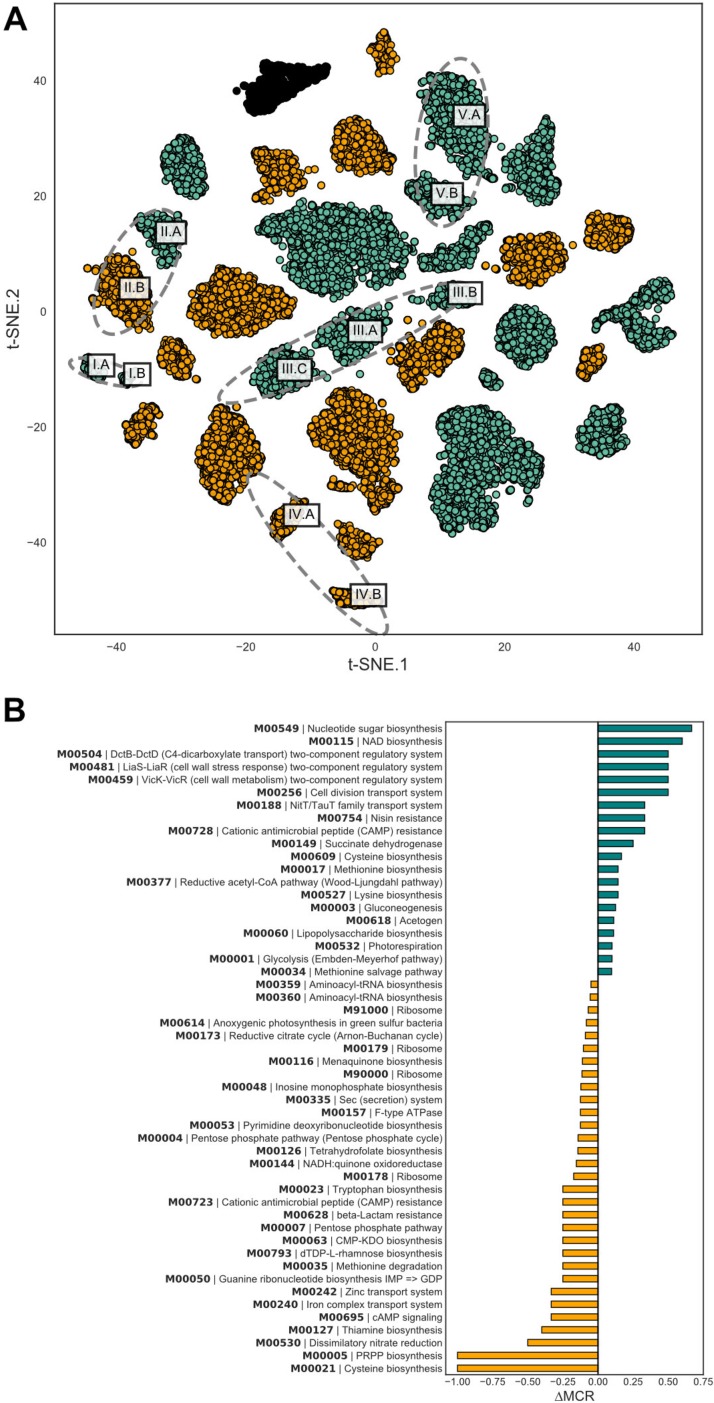
Metagenome assembled genomes recovered in assembly. Multiple MAGs of individual species and their functional differences. (A) t-SNE embeddings of center-log-ratio-transformed 5-mer profiles for each contig. Gold contigs have higher C and E coefficients in the ACE model distinguishing environmental acquisition while teal contigs indicate high A coefficients of heritable organisms. (B) Differences in functional potential of A. rava strains where one has a high heritability coefficient while the other has high environmentally associated coefficients according to the ACE model.

**FIG 6:**
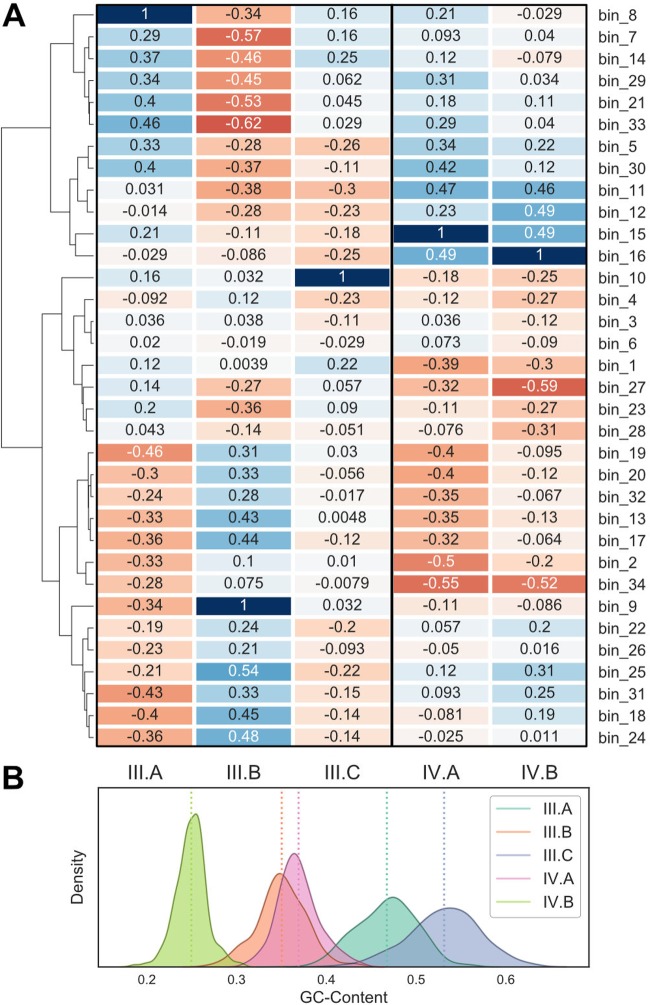
Supragingival microbial dark matter MAGs. TM7 and *Gracilibacteria* cooccurrence profiles and GC content distributions. (A) Spearman’s correlation for (left) TM7 and (right) *Gracilibacteria* abundance profiles against all other MAG abundance profiles. (B) GC content distributions for all contigs in corresponding MAGs from taxonomy groups III and IV.

The *k*-mer-based genome binning unveiled several closely related pairs or triplets of genomes (Fig. 5A). These include *Actinomycetes* (group I)*, Alloprevotella* (group II), “*Candidatus* Saccharimonas” TM7 (group III), *Gracilibacteria* (group IV), and *Prevotella* (group V) lineages (Table 1). This allowed us to examine the extent to which metabolic potential varies for ubiquitous microbial taxa in the human supragingival microbiome, two of which are from recently identified bacterial phyla (i.e., TM7 and *Gracilibacteria*). As shown in Fig. 5B, substantial variations in metabolic potential distinguish the different MAGs with emphasis on the *Alloprevotella* group. Similarly, the MAGs also display different abundances across subjects, cooccurrence patterns relative to other MAGs, and, in one case, heritability (Fig. 1A, 5A, and 6A).

The two *Alloprevotella* genomes recovered (denoted as MAGs II.A and II.B in Fig. 5A) contain one representative that has a high genetic component and another that is environmentally acquired, differing in the potential for nitrate reduction, biosynthesis of sulfur-containing amino acids, PRPP, and sugar nucleotides. *Alloprevotella* MAG II.B contains complete metabolic pathways for phosphoribosyl pyrophosphate (PRPP) biosynthesis from ribose-5-phosphate (M00005) and cysteine biosynthesis from serine (M00021); these metabolic modules are completely absent in the *Alloprevotella* MAG II.A. Similarly, the potential for dissimilatory nitrate reduction (M00530) is lacking in the heritable *Alloprevotella* MAG II.A but is present in MAG II.B.

Three MAGs for the recently discovered candidate phylum TM7 (32–34) were recovered, with each of them showing high heritability estimates. The recent cultivation of TM7 from the oral microbiome revealed a parasitic nature with *Actinobacteria* as hosts (34). With our recovered TM7 genomes, we observe very little cooccurrence patterns between TM7 MAG III.C and any other taxa. Curiously, TM7 MAGs III.A and III.B have an inverse relationship, in terms of cooccurrence profiles (*rho* = −0.34), suggesting different hosts or functional niches. TM7 MAG III.B is positively correlated with taxa associated with the caries-positive subjects—*Streptococcus*, C. morbi, and G. elegans—while MAG III.A is negatively associated with these taxa. TM7 MAG III.A contains complete pathways for dTDP-l-rhamnose biosynthesis (M00793), F-type ATPase (M00157), putative polar amino acid transport system (M00236), energy-coupling factor transport system (M00582), and SenX3-RegX3 (phosphate starvation response) two-component regulatory system (M00443) that are entirely absent in TM7 MAG III.B. TM7 MAGs III.A and III.B provide unique functional capabilities to the entire community, containing components for F420 biosynthesis (M00378) and SasA-RpaAB (circadian timing-mediating) two-component regulatory system (M00467), respectively.

Two MAGs with phylogenetic affinity to *Actinomyces* have been recovered in our analysis. *Actinomyces* MAG I.A exclusively has all of the components for the rhamnose transport system (M00220) while *Actinomyces* MAG I.B exclusively has components for the manganese transport system (M00316) compared to the rest of the community. Both *Actinomyces* MAG I.A and MAG I.B are the only genomes to contain components for the DevS-DevR (redox response) two-component regulatory system (M00482).

Two *Prevotella* sp. MAGs most similar to the Human Microbiome Project (HMP) oral taxon 472 (36) have been recovered in our analysis. Both *Prevotella* MAGs uniquely contain complete pathways or modules for CAM (crassulacean acid metabolism) (M00169), pyruvate oxidation via the conversion of pyruvate to acetyl-CoA (M00307), PRPP biosynthesis (M00005), beta-oxidation, acyl-CoA synthesis (M00086), adenine ribonucleotide biosynthesis via IMP-to-adenosine (di/tri)phosphate conversion (M00049), guanine ribonucleotide biosynthesis via IMP-to-guanosine (di/tri)phosphate conversion (M00050), coenzyme A biosynthesis from pantothenate conversion (M00120), cell division transport system (M00256), and putative ABC transport system (M00258).

Two MAGs for *Gracilibacteria* were recovered. Mapping of recent human oral WGS samples from the extended Human Microbiome Project (37) to these genomes show they are also found in North American subjects (Table S4). Metabolic reconstructions describe the capability for anoxic fermentation of glucose to acetate, as well as auxotrophies for vitamin B_12_ and a preponderance of type II and IV secretion systems. Both *Gracilibacteria* cooccurrence profiles show negative relationships with *Veillonella* sp. oral taxon 780 while showing positive relationships with Capnocytophaga gingivalis, suggesting competitive and commensal relationships, respectively. *Gracilibacteria* MAG IV.B is the only organism in our recovered oral community that has components for the BasS-BasR (antimicrobial peptide resistance) two-component regulatory system (M00451) and the cationic antimicrobial peptide (CAMP) resistance *arnBCADTEF* operon (M00721) while MAG IV.A exclusively contains components for the NrsS-NrsR (nickel tolerance) two-component regulatory system (M00464). *Gracilibacteria* MAG IV.A contains complete pathways for phosphate acetyltransferase-acetate kinase (M00579), the conversion of acetyl-CoA into acetate, while being completely absent in MAG IV.B. *Gracilibacteria* MAG IV.B contains complete components of the ABC-2 type transport system (M00254), which is completely absent in MAG IV.A.

10.1128/mBio.01631-18.6 TABLE S3 Variance component estimation results. Tabular-form representation of mets ACE model for MAGs and *Streptococcus* strains. Download Table S3, TXT file, 0.02 MB. Copyright © 2018 Espinoza et al. 2018 Espinoza et al. This content is distributed under the terms of the Creative Commons Attribution 4.0 International license.

10.1128/mBio.01631-18.7 TABLE S4 Extended Human Microbiome Project oral read mapping. Counts of reads from various oral sites in the extended HMP to our MAGs. Download Table S4, TXT file, 0.01 MB. Copyright © 2018 Espinoza et al. 2018 Espinoza et al. This content is distributed under the terms of the Creative Commons Attribution 4.0 International license.

## DISCUSSION

### Community-scale profiles for caries.

In this study, we took a hybrid approach of utilizing read mapping for taxa with excellent genome representation (e.g., *Streptococcus*) while generating new reference genomes *de novo* for less well represented organisms. These genome bins also allow for improved databases for future read mapping analyses and indeed identified taxa present in previous oral microbiome studies (37) that were overlooked. Our results suggest that caries status can be accurately associated with and, potentially, diagnosed by profiling specific taxa beyond Streptococcus mutans. We observed a loss of community diversity within the diseased subjects (Fig. 1C), a finding previously reported in 16S rRNA amplicon studies (21), with an increase in *Streptococcus* strain diversity (Fig. 2D). The observed differences in the overall community structure, as seen from our cohort study, suggest that profiling the abundance of multiple taxa may present an opportunity for caries diagnosis and preventative methods. This finding is also functionally relevant as many organisms, other than *Streptococcus,* statistically enriched within the caries-positive subjects are also both anaerobic glucose fermenters and acidogenic, a trend extending to strain-level *Streptococcus* populations as well. In the healthy cohort, the *Streptococcus* community exhibits less variation in terms of abundance and cooccurrence at the strain level. This is not the case in the diseased cohort, in which we observe higher Shannon entropy (Fig. 2). In an independent analysis, unsupervised clustering of diseased and healthy cohorts showed that ecological states in healthy subjects consistently have a *Streptococcus* community abundance that is either lower than or comparable to the rest of the healthy cohort (≤0.27), with the exception of cluster 7 (0.44), while being much more unpredictable in the diseased cohort (see Fig. S3 in the supplemental material). This phenomenon may be the result of grouping carious lesions of various degree into a single classification. However, this finding also supports the fact that caries is a dynamic and progressive disease whose rate of progression can change dramatically over short periods of time. These results suggest that caries onset cannot be described by a single bacterium but should be described as the perturbation of an entire ecosystem, consistent with hypotheses sourcing the disease from metabolic and community-driven origins.

10.1128/mBio.01631-18.1 FIG S1 Variance component estimation for MAGs. ACE model estimating the proportion of variance in a trait that is heritable versus the proportion from shared or unique environmental factors. (A) Stacked bar chart showing the proportions of A, C, and E components for each of recovered draft genomes bins using their scaled relative abundance as input. (B) ACE model estimates for *Streptococcus* strain clusters identified by MIDAS. Download FIG S1, EPS file, 1.1 MB. Copyright © 2018 Espinoza et al. 2018 Espinoza et al. This content is distributed under the terms of the Creative Commons Attribution 4.0 International license.

10.1128/mBio.01631-18.2 FIG S2 Cooccurrence profiles for MAGs. (A) Pairwise Spearman’s correlation of normalized abundance profiles. Red indicates negative cooccurrence relationships while blue indicates positive cooccurrence. (B) Swarm plots of cooccurrence magnitude represented by absolute value of Spearman correlation. Self-interactions were dropped from profiles. Download FIG S2, EPS file, 1.5 MB. Copyright © 2018 Espinoza et al. 2018 Espinoza et al. This content is distributed under the terms of the Creative Commons Attribution 4.0 International license.

10.1128/mBio.01631-18.3 FIG S3 Phenotype-specific ecological states. Dendrograms and heat maps of caries-positive and caries-negative cohorts for the core supragingival microbiome and *Streptococcus* species-level subsets. Bray-Curtis used for distance measures with Ward linkage for hierarchical clustering. (A) (Top) Healthy cohort and (middle) diseased cohorts clustered by MAGs separately. (Bottom) Heat map of relative abundance for each MAG. (B) (Top) Healthy cohort and (middle) diseased cohorts clustered by *Streptococcus* species separately. (Bottom) Heat map of relative abundance for each *Streptococcus* species. Download FIG S3, EPS file, 1.3 MB. Copyright © 2018 Espinoza et al. 2018 Espinoza et al. This content is distributed under the terms of the Creative Commons Attribution 4.0 International license.

The dynamics of the *Streptococcus* community, at both the genus and strain levels, explain the observation of low connectivity in the diseased cooccurrence network, supporting our hypothesis that caries onset is a community perturbation. The highly connected cluster in the microbial community cooccurrence network (Fig. 2C) can be interpreted as influential organisms that drive the dynamics of other taxa in the community. The aforementioned high-PageRank microbial clique can be subdivided into a subset enriched in diseased subjects (Fig. 3, cluster 3) and a subset enriched in healthy individuals (Fig. 3, cluster 4). It is possible that disease state is influenced by a balance between these microbes, though empirical *in situ* studies are required to move past speculation.

### Caries is a community-scale metabolic disorder.

Our metabolic and community composition analyses challenge a single-organism etiology for caries and coincide with previously published ecological perspectives (19, 23, 25, 26, 38, 39). These authors suggested that the changes in functional activities of the microbiome were the major cause of caries while simultaneously suggesting regime shifts (e.g., see reference 40) in community composition. They posited that increases in sugar catabolism potential are the key markers of caries progression; here we observed enrichments in nearly a dozen phosphotransferase sugar uptake systems. A novel insight was the enrichment for diverse sugar uptake pathways instead of just those associated with glucose, notably, glucose, galactitol, lactose, maltose, alpha-glucoside, cellobiose, and *N*-acetylgalactosamine phosphotransferase uptake pathways were all enriched. Takahashi and Nyvad (7) proposed an “extended ecological caries hypothesis,” suggesting that community metabolism regulates adaptation and selection; once acid production starts to proceed, it provides evolutionary selection for adaptations to variable pH. Analogous to the expansion of the S. mutans-centric paradigm to the entire community, the structural potential of the community for sugar catabolism diversifies greatly in caries-positive states. This suggests that a healthy phenotype has self-stabilizing functional potential; numerous sugar compounds are not easily taken up by the community, preventing the associated pH decrease. In contrast, the progression to a caries state increases the diversity of sugar compounds available to the community catabolic network, thereby facilitating a pH decrease from an increased proportion of dietary input. This likely leads to increased pH fluctuations in both frequency, magnitude, and, potentially, duration. This is entirely consistent with the hypothesis that caries dysbiosis results from a community-scale metabolic shift and that progression has a feed-forward evolutionary adaptation pressure. At this point, it is difficult to identify the originating environmental pressure that might positively select for catabolism of diverse sugars, though host diet is a possible explanation.

The increase in sugar uptake potential is unsurprising but also increases confidence in the other trends that have not been described before. Within caries-positive microbiomes, we observed a notable enrichment of numerous two-component histidine-kinase response-regulator pairs, which are responsible for transcriptional changes in response to environmental stimuli. It is likely that this represents a community adaptation to increased variations in biofilm pH due to increased sugar catabolism, as well as a more dynamic community-scale microbial interaction network. Pathways for antibiotic resistance were also enriched in the communities of caries-positive subjects. To our knowledge this has not been previously reported. However, this could be an artifact of database bias, considering that carious lesions are associated with enrichment of *Streptococcus*, which has an abundance of well-characterized antibiotic resistance gene systems. An alternative explanation leverages the observation that carious states are characterized by inherently chaotic community profiles. Specifically, healthy caries-free states are associated with a relatively stable *Streptococcus* community, whereas the caries-positive *Streptococcus* communities are far more dynamic and diverse (Fig. 2D). It is possible that increased pH temporal gradients associated with caries select for antibiotic resistance due to increased antagonistic microbial interactions. The possibility that antibiotics included in toothpaste influence the prevalence of antibiotic resistance pathways could not be tested here.

The most surprising and difficult-to-explain enrichment in community functional potential associated with caries is the gain of metal transport pathways involved in the efflux of Zn or Mn, Ni, and Co. This is not likely due to a host response, as this normally involves host acquisition of Fe, Mn, and Zn, along with Cu efflux (41–43). We propose that the degradation of enamel, and subsequently dentin, results in the release of locally toxic levels of trace metals for the biofilm constituents, thereby providing an environmental selection pressure for the detoxification of these elements. This parallels geomicrobiology surveys where microbial activity degrades physical substrates like rocks and inorganic minerals (44). It also follows that other governing principles based on geomicrobiology systems may apply to the human supragingival microbiomes.

### Functional plasticity within poorly described core supragingival microbiome lineages.

We often ascribe functional potential to species based on type strains. To some extent, this is sensible as function and phylogeny are interlinked, and this has resulted in computational tools that associate phylogenetic loci, such as 16S rRNA, with functional potential (45). However, the genome plasticity makes it difficult to extrapolate function with confidence in some cases. For example, it has been shown that some strains of S. mutans can be less acidogenic than other streptococcal species (46), which could affect the organism’s pathogenicity in the context of cariogenesis. This phenomenon may serve to explain why we did not witness an enrichment of S. mutans in the diseased microbiota, an observation noticed in previously published studies (47–49). Our genome-centric approach revealed rather dramatic differences in functional potential between MAGs in the core supragingival microbiome. These include changes in vitamin and amino acid auxotrophy, environmental sensing, and nitrate reduction. Closely related genomes also exhibited differences in patterns of abundance and cooccurrence across the 88 subjects. The differences in functional potential and genome autecology suggest these taxonomically similar microbes have different metabolic roles in the supragingival microenvironment as mentioned in previous microbiome studies from the gut consortia (50) and *in vitro* biofilm cultures (51). Many of the newly described core supragingival microbiome MAGs greatly increase our genomic knowledge about previously poorly described lineages. In particular, to date only two *Alloprevotella* genomes have been published, only one TM7 oral microbial genome has been described, and this is the first description of oral *Gracilibacteria*. In all cases, multiple genomes with different autecology and metabolic capabilities were recovered.

### Alloprevotella.

The type strain, Alloprevotella rava, historically referred to as Bacteroides melaninogenicus, is an anaerobic fermenter producing low levels of acetic acid and high levels of succinic acid as fermentation end products while being weakly to moderately saccharolytic (52). Only two reference genomes are available for *Alloprevotella*, including A. rava and *Alloprevotella* sp. oral taxon 302 (37). As mentioned above, our analysis recovered two MAGs of A. rava, one of which has a high genetic component (MAG II.A; A = 0.64) and the other of which is environmentally acquired (MAG II.B), according to the ACE model. The environmentally acquired *Alloprevotella* MAG II.B has reduced potential for cysteine biosynthesis, specifically the conversion of methionine to cysteine (M00609), and appears to be a methionine auxotroph; the potential for methionine biosynthesis from aspartate (M00017) and the methionine salvage pathway (M00034) are both higher in MAG II.A (Fig. 5B). Furthermore, *Alloprevotella* MAG II.A is completely lacking all the components for cysteine biosynthesis from serine, but can synthesize cysteine from methionine. The amino acid auxotrophies can be satisfied by the amino acids found in saliva, but the divergence in cysteine biosynthesis pathways is striking. A similar scenario exists for cationic antimicrobial peptide (CAMP) resistance: *Alloprevotella* MAG II.A contains more envelope protein folding and degradation factors (e.g., DegP and DsbA) while phosphoethanolamine transferase EptB is found in the environmentally acquired MAG II.B. Interestingly, the heritable strain contains more components for two-component regulatory systems (M00504, M00481, and M00459), transport systems (M00256 and M00188), and nisin resistance (M00754) than environmentally acquired MAG II.B. A final key metabolic acquisition novel to *Alloprevotella* MAG II.B relative to both cultivated and uncultivated *Alloprevotella* is the presence of components necessary for dissimilatory nitrate reduction. The oral production of nitrite from dietary or saliva-derived nitrate is the first step in the enterosalivary nitrate circulation (16), and this is the first implication of *Alloprevotella* in this cycle. The environmentally acquired *Alloprevotella* MAG II.B has a larger genome with a slightly higher CheckM-calculated contamination than MAG I.A, suggesting that there may be several Alloprevotella rava strains that are acquired by the environment with similar *k*-mer content. Interestingly, *Alloprevotella* MAG II.B is also enriched in enamel caries compared to caries that have progressed to the dentin layer, indicating that the environmentally acquired strains may play a role in the onset of carious lesions. Longitudinal metagenomic studies would be necessary to determine if the environmentally acquired Alloprevotella rava displaces the heritable strain with age and diet.

### TM7.

TM7 has been found in a variety of environments using cultivation-independent methods such as 16S rRNA sequencing. The first described genomes were assembled from wastewater reactor (53) and groundwater aquifer metagenomes, while the first cultivated strain was derived from an oral microbiome (34). A comparison of those three genomes revealed highly conserved genomic content and, generally, low percent GC (34). The recovery of three TM7 MAGs (named oral_TM7_JCVI III.A, III.B, and III.C) greatly adds to our knowledge about this unique lineage. Even the most coarse-grained comparative genomics analysis, that is, average GC content, revealed that MAGs III.A, III.B, and III.C are quite divergent (Fig. 6). TM7 MAG III.B is most similar to the cultivated strains at 35% GC, while MAG III.A is 46% GC and MAG III.C is 55% GC. TM7 MAG III.A is almost certainly a pangenome of multiple TM7 strains of relatively intermediate GC content based on the bimodal GC profile and the CheckM contamination of 159.28, though they were clearly separated in 5-mer space (Fig. 5). TM7 MAG III.B has an inverse cooccurrence pattern with MAG III.A (*rho* = −0.34) and no cooccurrence relation with MAG III.C (*rho *=* *0.03) in this study (Fig. 6A), strongly suggesting they have different ecological roles. Altogether, these TM7 genomes likely each represent a new family within the TM7 phylum.

The cultivated TM7 is an obligate epibiont parasite of oral *Actinomyces* (34). However, we did not observe strong cooccurrence patterns between the TM7 MAGs and any of the *Actinobacteria,* though it is not known if these relationships should have a strong cooccurrence or a lagged predator-prey cycle. The TM7 MAG III.B genome contains a near-complete pentose phosphate pathway, which indicates that this MAG has gained at least one form of energy-generating sugar metabolism relative to the other TM7. The TM7 MAG III.A genome uniquely contains the pathway for the synthesis of cofactor F420, though none of the components for methanogenesis. Instead, it is likely a cofactor in a flavin oxidoreductase of unknown function (54) and possibly involved in nitrosative (55) or oxidative stress (56). Given the likely production of nitrite by *Alloprevotella* and other members of the supragingival biofilm via dissimilatory nitrate reduction, the ability to detoxify nitrite has a protective role for both TM7 MAG III.A and its putative host.

### *Gracilibacteria*.

The presence of *Gracilibacteria* in the human oral microbiome was first reported in 2014 through 16S rRNA screening, with two 16S clades from human oral or skin samples (33). The first genome for *Gracilibacteria* was a single amplified genome from a deep-sea hydrothermal environment (57), and here we describe two *Gracilibacteria* MAGs that, analogous to TM7, are differentiated by fundamental genomic characteristics. *Gracilibacteria* MAG IV.A has a percent GC of around 37% while MAG IV.B averages 25% GC, making it one of the lowest-GC genomes reported (Fig. 6B). Both *Gracilibacteria* MAGs use the alternative *opal* stop codon. The *Gracilibacteria* MAGs detected here are most phylogenetically similar to those recovered from the East Central Atlantic hydrothermal chimney (57, 58). Both of our recovered *Gracilibacteria* MAGs appear to be anaerobic glucose fermenters producing acetate as a product, while containing numerous putative auxotrophies for vitamins and amino acids (see Table S5 in the supplemental material). Each genome bin contains at least two secretion systems (type II and IV) and CRISPR/CAS9 systems. Based on the small genomes, low GC content, and limited metabolic potential, we propose that these organisms are likely intracellular, or epibiont, parasites of other bacteria analogous to TM7. The two oral *Gracilibacteria* differ moderately with regard to functional potential and genome autecology. *Gracilibacteria* MAG IV.B contains an enrichment in functional potential for cationic antimicrobial peptide (CAMP) resistance (M00721, M00722, and M00728) and two-component pathways suggesting more metabolic interactions than MAG IV.A. *Gracilibacteria* MAG IV.B also uniquely contains an ABC-2-type polysaccharide or drug efflux system. *Gracilibacteria* MAG IV.A is also significantly enriched in healthy individuals, suggesting that this MAG may be beneficial for maintaining stable microbial solution states with regard to caries status.

10.1128/mBio.01631-18.8 TABLE S5 Module completion ratios from MAPLE. MCRs for each of our MAGs. Download Table S5, TXT file, 0.1 MB. Copyright © 2018 Espinoza et al. 2018 Espinoza et al. This content is distributed under the terms of the Creative Commons Attribution 4.0 International license.

*Gracilibacteria* MAGs IV.A and IV.B strongly cooccur with one another, suggesting a commensal or synergistic relationship. *Gracilibacteria* MAG IV.A has negative cooccurrence with *Veillonella* sp. oral taxon 780 and *Actinomyces* MAG I.B (*rho* = −0.549 and −0.497, respectively) with a positive cooccurrence with Capnocytophaga gingivalis and *Prevotella* MAG V.B (*rho *=* *0.467 and 0.422, respectively). *Gracilibacteria* MAG IV.B has negative cooccurrence with Prevotella oulorum and *Veillonella* sp. oral taxon 780 (*rho* = −0.589 and −0.516, respectively) with a positive cooccurrence with Cardiobacterium hominis and Capnocytophaga gingivalis (*rho *=* *0.486 and 0.462, respectively). *Veillonella* plays a role in the anaerobic fermentation of lactate to propionate and acetate by the methylmalonyl-CoA pathway. As mentioned above, Capnocytophaga gingivalis is found along the *Corynebacterium* base of the biofilm but in high density within the annulus due to a high demand for carbon dioxide (59). This may suggest that *Gracilibacteria* resides in the annulus of the biofilm, moderately interacting with *Corynebacterium* in regions where carbon dioxide is accessible. The enrichment in cooccurrence of *Gracilibacteria* MAG IV.B with Neisseria oralis compared to MAG IV.A (*rho *=* *0.314 and 0.122, respectively) may suggest that MAG IV.B colonizes the biofilm periphery in microaerophilic environments.

### Conclusions.

Collectively, we have provided a holistic overview of the juvenile supragingival plaque microbiome. Many of the same genomes were found across all 88 subjects, describing a core microbiome. With the presence of caries, the abundance of constituents of the core microbiome changes to a variety of ecological states where the community networks are perturbed. The phenotypes of health and carious lesions should be characterized not only by the abundance of taxa but also by the functional potential of the community. Sugar-fermenting bacteria are always enriched in carious states, as are the abundance and diversity of sugar uptake pathways. This strongly supports the idea that caries phenotype is a community metabolism disorder. Carious lesions are also accompanied with an enrichment in environmental sensing and antibiotic resistance, suggesting that acid production provides a selection pressure. Finally, we provide genomic information about the metabolic diversity of several organisms represented by poorly described microbial lineages.

## MATERIALS AND METHODS

### Study design.

Our objective was to compare the metagenomic signatures, in dental plaque, of children with and without dental caries. Our hypotheses were that (i) there would be a measurable difference in species and diversity between the two groups and (ii) some species present in plaque samples associated with caries will have been previously implicated in cariogenesis. Dental plaque samples were collected from participants of the University of Adelaide Craniofacial Biology Research Group (CBRG) and the Murdoch Children’s Research Institute (MCRI)’s Peri/Postnatal Epigenetic Twins Study (PETS) (60). The PETS (*n* = 193) and CBRG (*n* = 292) cohorts were composed of twins 5 to 11 years old. Twins from the state of Victoria, Australia (PETS), were recruited during the gestation period. All contactable twins from the PETS cohort will be eligible for participation. Inclusions were those twins whose parent consented to this particular wave of the study and who were recruited into the study during the gestation period. Ethics approval was attained from the University of Adelaide (Adelaide, Australia) Human Research Ethics Committee, The Royal Children's Hospital Melbourne (Parkville, Australia) Human Research Ethics Committee, and the J. Craig Venter Institute Institutional Review Board.

Dental plaque samples were obtained at the commencement of a dental examination. The participants had not brushed their teeth the night preceding the plaque collection and on the day of collection. Additional data were collected from three separate questionnaires completed by the parents during the period from consent to prior to the dental examination being undertaken. The combined questionnaires consisted of 132 questions regarding oral health, dietary patterns and general health and development. Fifteen of these were extracted for use by JCVI for this analysis.

The entire dentition of each participant was assessed using the International Caries Detection and Assessment System (ICDAS II) (61). The ICDAS II is used to assess and define dental caries at the initial and early enamel lesion stages through to dentinal and final stages of the disease. Examiners were experienced clinicians who had undergone rigorous calibration and were routinely recalibrated across measurement sites to minimize error. Caries experience in each participant was initially reduced to a whole-mouth score, and three classifications were utilized: no evidence of current or previous caries experience, evidence of current caries affecting the enamel layer only on one or more tooth surfaces, and evidence of previous or current caries experience that has progressed through the enamel layer to involve the dentin on one or more tooth surfaces (including restorations or tooth extractions due to caries). For the purpose of this analysis, we classified disease phenotypes in twins as presence of caries in enamel or dentin.

The number of pairs selected for metagenomic sequencing was constrained by budget; thus, a subset of the broader clinical cohort was subsampled. Twin pairs were selected for sequencing manually by examining ordination plots from the broader 16S rRNA gene sequencing study (27) and then selecting (i) twins of the same phenotype that were closely related and (ii) twins discordant for caries that were divergent in ordination space.

### Sample collection, DNA extraction, library prep, and sequencing.

Plaque sample collection and DNA extraction were conducted as specified in reference 27. Libraries were prepared using the NEBNext Illumina DNA library preparation kit according to manufacturer’s specifications (New England Biolabs, Ipswich, MA). Metagenomic libraries were sequenced using the Illumina NextSeq 500 High-Output kit for 300 cycles following standard manufacturer’s specifications (Illumina Inc., La Jolla, CA). Subjects without caries in enamel or dentin are referred to as healthy while subjects with caries in either enamel or dentin are referred to as diseased unless otherwise noted.

### Metagenomic coassembly.

Kneaddata (62) was used for quality trimming on the raw sequencing reads, as well as screening out host-associated reads. Assembly was performed using SPAdes genome assembler v3.9.0 (metaSPAdes mode) (63) with the memory limit set to 1,024 GB. Reads were pooled, and initial assembly attempts resulted in exceeding memory limits. Therefore, each library was subsampled randomly to 25% of the total for the final coassembly. Quality-trimmed reads from all samples were mapped back to the final metagenomics coassembly to generate abundance profiles across all 88 subjects.

### Genome binning.

The t-Distributed Stochastic Neighborhood Embedding algorithm (64, 65) applied to center-log-transformed *k-*mer profiles (*k *=* *5) implemented in VizBin (66) raises a memory error when computing metagenomics assemblies of this scale. To address this issue, we developed a semisupervised iterative linear algebra technique to extract metagenome assembled genomes (MAGs) from the deluge of contigs in the *de novo* assembly. The pipeline is as follows: (step 1) use a conservative contig size threshold of 2,500 nucleotides for the initial binning; (step 2) eigendecomposition to calculate a representative vector in the direction of greatest variance (i.e., the 1st principal component [PC1]) of (2a) the coverage profiles and (2b) the *k-*mer profiles for each visually identified bin (66) of larger contigs; (step 3) subset the remaining contigs between the bandwidth of 300 and 2,500 nucleotides while computing the Pearson correlation between each bin’s PC1 and each contig in the smaller-contig-length subset; (step 4) extract the contigs (*n* = 500 contigs per bin) with the highest Pearson correlation to each bin’s PC1 from steps 2a and 2b; (step 5) merge the results of step 4 with the coarse bins from step 1 and recalculate the embeddings to generate finer-scale bins; and (step 6) iterate step 5 until convergence to yield the finalized draft genome bin (MAG). The quality of each of the finalized draft genome bins, or MAGs, was assessed for completeness, contamination, and strain-level heterogeneity using CheckM (v1.07) (29). Streptococcal annotated contigs were set aside during the binning process due to their promiscuous *k*-mer usage between strains.

### Annotation.

Annotation methods were as described in reference 67 with slight modifications. Open reading frames (ORFs) were called with FragGeneScan (v1.16), except the *Gracilibacteria* bins, which were called with Prodigal (v2.6.3), using the Candidate Division SR1 and *Gracilibacteria* genetic code (trans_table = 25) (68, 69). For ORF annotations, domains were characterized using a custom compilation database with HMMER (v3.1b2) and functionality was further assessed via best-hit BLAST results and manual curation (70, 71). Contig and draft genome bin taxonomic assignments were determined by calculating a running sum of percent amino acid identities of the ORFs for each bin grouped by their respective taxonomy identifier from PhyloDB (https://github.com/allenlab/PhyloDB). PhyloDB is a comprehensive database of existing NCBI genomes, JGI-only single amplified genomes, and the MMETSP (72). The maximum weighted taxon at the species level was used to classify each MAG. The phylogenetic tree traversal was conducted using ete3’s NCBITaxa object, implemented in Python, to extract labeled hierarchies from taxonomic identifiers (73).

### Microbial community composition.

Metagenomic reads were mapped to contigs using CLC with a minimum spatial coverage of 50% and a minimum percent identity of 85%. The resulting count tables were transformed by adapting the transcript per million (TPM) (74) calculation for use on contigs to incorporate length, coverage, and relative abundance into the measure, an essential normalization for metagenome assemblies due to the inherently wide distribution of contig lengths. Summations of TPM values grouped by bin assignment were used as abundance values for draft genome composition and downstream analysis unless otherwise noted.

Strain-resolved *Streptococcus* abundances were calculated using the Metagenomic Intra-Species Diversity Analysis System (MIDAS) (5) with the subsampled subject-specific reads. MIDAS subprograms “genes” and “species” were run with default settings of 75% alignment coverage, 94% percent identity, and mean quality greater than 20. *Streptococcus* strains were parsed from the subject-specific count matrices to build a master count matrix containing all *Streptococcus* strains with respect to each individual twin.

### Statistical analyses.

Pairwise log_2_ fold change (logFC) profiles were calculated between groups by combinatorically computing the logFC between each nonredundant pair of the comparison groups and taking the mean of the distributions. All *P* values were calculated using the Mann-Whitney U test unless specifically noted otherwise. The statistical significance values were set at an inclusive threshold of 0.05 for determining enrichment in the following contexts: (context 1) MAGs via microbial abundance between diseased and healthy phenotypes, (context 2) functional module via phylogenomically binned functional potential (PBFP) profiles (see below) between diseased and healthy phenotypes, and (context 3) functional modules via PBFP profiles that were statistically significant between the groups identified in context 1. Spearman’s correlation coefficients are denoted as *rho* unless otherwise noted.

### Cooccurrence network analysis.

Pairwise Spearman’s correlations were used to robustly measure monotonic relationships between microbial abundance profiles, calculated for the MAGs and *Streptococcus* strains separately. MAG abundance profiles were standardized by TPM normalization, log transformation, and z-score normalization for each subject. In the *Streptococcus* strain-level cooccurrence analysis, strains were dropped from the calculation if they were not present in at least 10% of the samples. To account for domain errors during log transformation, due to zero values in the *Streptococcus* strain-level counts matrix, a pseudocount of 1e−4 was added to the entire dataframe. The WGCNA R package was used to calculate the adjacency and topological overlap measures (TOM) of the weighted cooccurrence network (75, 76). The TOM similarity matrix was used to construct the fully connected undirected NetworkX graph structure (77). The dendrogram visualizations were constructed from the dissimilarity representation of the TOM matrix (i.e., 1 − TOM_Similarity_) using ward linkage in SciPy (v1.0). PageRank centrality values (35) were computed on the fully connected weighted networks using the implementation provided in NetworkX. PageRank centrality is a variant of eigenvector centrality which allows us to measure the influence of bacterial nodes within our cooccurrence networks. We implemented PageRank centrality because it can be applied to fully connected weighted networks while many other, more common measures (e.g., Katz centrality) are not applicable in this setting. All analyses were conducted in Python (v3.6.4), and figures were generated using Matplotlib (v2.0.2) unless otherwise noted (78).

### Variance component estimation.

Assessing the additive genetic and environmental factors driving MAG abundance as determined by the ACE model (79), controlling for sex, age, and health phenotype, was described previously in reference 27. The ACE model assumes that the variability of a given attribute is explained by additive (A) genetic effects, the shared/common (C) environment, and nonshared/unique environmental (E) factors. To these ends, the MAG abundance data were standardized by the following procedure: (i) calculating proportions of each bin (i.e., relative abundance of summed TPM values per bin); (ii) log transformation of normalized microbial abundances; and (iii) z-score normalization for each draft genome bin. The ACE model for variant component estimation was implemented using the mets package in R (80).

### Functional potential profiling.

To determine the functional components of MAG, we translated ORFs for each bin to build a putative proteome. The University of Kyoto’s Metabolic And Physiological potential Evaluator (MAPLE v2.3.0) (81, 82) was used to compute the Module Completion Ratios (MCRs) representing KEGG pathways, complexes, functions, and specific signatures. The MCR is calculated using a Boolean algebra-like equation previously described in reference 82. In order to identify latent interactions between these KEGG modules, MAGs, and specific subjects, we developed a metric that incorporates genome coverage, proportion, and MCRs referred to from this point forward as Phylogenomically Binned Functional Potential (PBFP). With this measure, we were able to investigate differences in functional modules within the context of subject metadata (e.g., caries status). Subject-specific PBFP profiles were computed by summing matrix *D* (*n* = subjects, *m* = contigs) across the contig axis with respect to draft genome bin assignment to produce matrix *X* (*n* = subjects, *p* = MAGs). Matrix multiplication was computed for the MCRs in matrix *C* (*p* = MAGs, *q *= modules) and the abundance measures in *X* to yield a transformed matrix *A* (*n* = subjects, *q* = modules) with subject-specific PBFP profiles for subsequent analysis.

### Data availability.

New methods are available at https://github.com/jolespin/supragingival_plaque_microbiome. All reads and assemblies are available in BioProject PRJNA383868. The overall coassembly is available through biosample SAMN10133834. The genome bines are available through biosample accession numbers SAMN10134551 to SAMN10134584. The individual reads for each library are available through accession numbers SRR6865436 to SRR6865523.
